# Preventative effects of ramelteon against postoperative delirium after elective liver resection

**DOI:** 10.1371/journal.pone.0241673

**Published:** 2020-11-02

**Authors:** Daisuke Hokuto, Takeo Nomi, Takahiro Yoshikawa, Yasfuko Matsuo, Naoki Kamitani, Masayuki Sho

**Affiliations:** Department of Surgery, Nara Medical University, Kashihara-shi, Nara, Japan; UKSH Campus Lübeck, GERMANY

## Abstract

**Background:**

Postoperative delirium was reported to be associated with increased postoperative mortality after liver resection. Therefore, it is crucial to prevent postoperative delirium in such cases. Ramelteon, an agonist of melatonin receptor has been suggested to be useful for preventing delirium. The aim of this study was to examine whether ramelteon is effective at preventing delirium after elective liver resection.

**Methods:**

The cases of patients who underwent liver resection at Nara Medical University (Nara, Japan) between January 2014 and August 2018 were analyzed. During the period from January 2017 to August 2018, ramelteon was prospectively administered to patients who underwent liver resection [8 mg/day on the day before surgery and on postoperative days 1 to 3] (ramelteon group), whereas ramelteon was not administered during the period from January 2014 to December 2016 (control group). The perioperative outcomes of the two groups were compared.

**Results:**

There were 120 patients in the ramelteon group and 186 patients in the control group. No significant intergroup differences in background factors, including age, gender, and preoperative serological laboratory data, were detected. The incidence of postoperative delirium was significantly lower in the ramelteon group (5.8% vs. 15.1%, P = 0.035). Multivariate analysis revealed that being aged ≥75 (P = 0.002), being male (P = 0.020), cardiovascular disease (P = 0.023), blood loss ≥1000ml (P = 0.001) and the absence of ramelteon treatment (P = 0.046) were independent risk factors for postoperative delirium.

**Conclusion:**

The administration of ramelteon might reduce the risk of postoperative delirium after elective liver resection.

## Introduction

Postoperative delirium is a common complication after surgery. It often leads to a prolonged recovery time, increased rates of other complications, a requirement for extra nursing care, and higher medical costs. Furthermore, an association between postoperative delirium and increased postoperative mortality was reported to exist [1, 2]. Therefore, there is a need for strategies for preventing postoperative delirium. Regarding liver resection, postoperative delirium was reported to be an independent risk factor for a poor prognosis [3]. A few studies have reported risk factors for postoperative delirium after liver resection [4–6]. However, no previous study has reported a strategy for preventing postoperative delirium after liver resection.

It has been suggested that ramelteon, a melatonin agonist that is used to treat insomnia, might be useful for preventing delirium. A randomized controlled trial suggested that the administration of ramelteon to elderly patients who are admitted for acute care might provide protection against delirium [7, 8]. Moreover, some retrospective studies have demonstrated that ramelteon is effective at preventing postoperative delirium after thoracic surgery [9, 10]. On the other hand, one RCT reported no significant effect for preventing postoperative delirium after elective cardiac surgery [11]. However, recent meta-analysis suggested the efficacy of ramelteon against postoperative delirium [12, 13]. Based on these findings, the perioperative administration of ramelteon to prevent postoperative delirium was considered in our department. Since January 2017, ramelteon has been routinely administered during the perioperative period to patients who undergo hepatectomy at our hospital. The aim of this study was to examine whether ramelteon is effective at preventing delirium after elective liver resection.

## Materials and methods

### Study population

The study protocol was approved by the ethics committee of Nara Medical University (No. 1991), and the study was registered at the UMIN Clinical Trials Registry (R000040659). Written informed consent for clinical research was performed before liver resection. Notification of opt-out for this study was displayed in the website of our department.

The data for all consecutive patients who underwent liver resection at Nara Medical University (Nara, Japan) between January 2014 and August 2018 were retrieved from a prospective database for this retrospective study. During the period from January 2017 to August 2018, ramelteon was routinely administered to patients who underwent liver resection [8 mg/day on the day before surgery and on postoperative days (POD) 1 to 3] (the ramelteon group), whereas ramelteon was not administered during the period from January 2014 to December 2016 (the control group). The patients of the rameleton group and the patients of the control group underwent same perioperative management except for administration of ramelteon. The perioperative outcomes of the two groups were compared. Patients who underwent biliary reconstruction or developed ileus were excluded because these patients could not take ramelteon on POD1.

### Preoperative assessments

Various preoperative assessments, including blood biochemistry tests, percutaneous ultrasonography, computed tomography, magnetic resonance imaging of the liver, and routine cardiorespiratory evaluations, were performed. The patients’ medical history, comorbidities, and American Society of Anesthesiologists (ASA) scores were recorded. Liver function was assessed using both the Child-Pugh score and the indocyanine green (ICG) clearance test.

### Surgical procedures

All resection procedures were performed with curative intent. Major hepatectomy was defined as the resection of more than three contiguous segments, according to Couinaud’s classification. Anatomical resection was defined as segmentectomy, sectionectomy, hemi-hepatectomy, or tri-sectionectomy. Wedge resection was defined as any non-anatomical resection. All liver resection procedures were performed via a laparotomic or laparoscopic approach. Various intraoperative parameters, including blood loss, blood transfusion use, and the duration of surgery, were recorded.

### Postoperative outcomes

Parameters associated with postoperative liver function (i.e., serum liver transferase and bilirubin levels) were measured on POD 1, 3, 5, and 7. Postoperative complications were stratified according to the Clavien-Dindo classification. Major complications were defined as those of grade IIIa or higher. Bile leakage was defined as grade B/C according to the International Study Group of Liver Surgery (ISGLS) classification. Surgical site infections were diagnosed according to the Centers for Disease Control and Prevention guidelines [14]. Liver failure was defined as grade B/C according to the ISGLS classification.

### Evaluation of postoperative delirium

Each patient who underwent liver resection was prospectively evaluated for postoperative delirium by the attending surgeons and bedside nurses. Evaluation was performed by a conference at every morning and evening. This study was focused on postoperative delirium which caused interruption of normal postoperative care. Therefore, in this study, postoperative delirium was defined as the status that normal postoperative care was interrupted due to problems with the patient’s level of consciousness or behavior. Delay of ambulation or oral intake, using extra psychotropic drug for delirium, and using extra behavior monitoring system were regarded as postoperative delirium. Delirium was also evaluated by the American Psychiatric Association’s fifth edition of the Diagnostic and Statistical Manual of Mental Disorders (DSM-5). DSM-5 identifies delirium by following 5 factors. A: Disturbance in attention and awareness, B: The disturbance develops over a short period of time, C: An additional disturbance in cognition, D: The disturbances in Criteria A and C are not better explained by a pre-existing, established or evolving neurocognitive disorder and do not occur in the context of a severely reduced level of arousal such as coma, E: There is evidence from the history, physical examination or laboratory findings that the disturbance is a direct physiological consequence of another medical condition, substance intoxication or withdrawal. Therefore, we have not diagnosed the patients with obvious hepatic coma or pre-existing mental disorder as postoperative delirium. Delirium was classified as hyper active type, hypoactive type, and mixed type according to DSM-5. The postoperative day (POD) when the delirium developed, duration of the delirium, and treatment of the delirium were recorded.

### Statistical analyses

Continuous data are expressed as median and range values. Qualitative variables are expressed as frequencies (percentages). The Student’s *t*-test or the Mann-Whitney *U* test was used for intergroup comparisons of quantitative variables as appropriate, whereas the χ^2^ test or Fisher’s exact test was used to compare categorical data. Two-sided p-values of <0.05 were considered to be statistically significant. All statistical analyses were performed using SPSS for Windows version 22.0 (SPSS, Inc.).

## Results

### Patients

**Fig 1:**
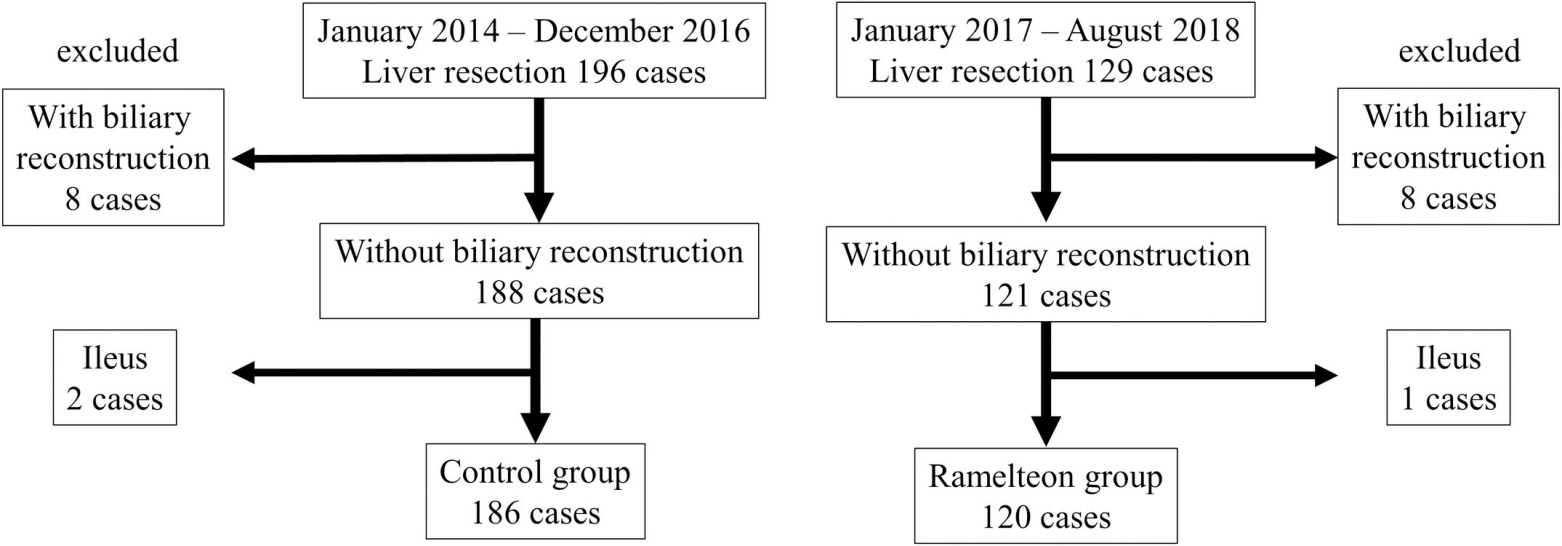
A flow chart of the eligible/included patients is shown.

Between January 2014 and August 2018, 325 patients underwent liver resection consecutively at Nara Medical University hospital. Of these, 196 patients were treated between January 2014 and December 2016, while 129 patients were treated between January 2017 and August 2018. The patients who underwent biliary reconstruction or developed ileus were excluded. As a result, 120 patients were administered ramelteon (the ramelteon group), whereas 186 were not (the control group) (Fig 1).

### Baseline characteristics

Of the 306 patients enrolled in the present study, 210 (68.6%) were male, and 96 (31.4%) were female. Their median age was 70 years (range, 30–88). One hundred and fifty-four (50.3%) patients were diagnosed with hepatocellular carcinoma (HCC), while 86 (28.1%) patients were diagnosed with colorectal liver metastasis. The age and gender distributions and the frequencies of preoperative complications did not differ significantly between the two groups. The preoperative serological laboratory data of the two groups, including their albumin, total bilirubin, and creatinine levels and their ICG retention rate at 15 minutes (ICG-R15) values, were similar (**Table 1**).

**Table 1:** Baseline characteristics.

	ramelteon n = 120	control n = 186	P-value
Age, median (range)	71 (34–85)	69 (30–88)	0.269
Gender, male, n (%)	75 (62.5)	135 (72.6)	0.064
Present illness, n (%)			
Hepatocellular carcinoma	61 (50.8)	93 (50.0)	0.887
Colorectal liver metastases	39 (32.5)	47 (25.3)	0.170
Other	20 (16.7)	46 (24.7)	0.094
Alcohol, n (%)	15 (12.5)	24 (12.9)	0.918
Viral status, n (%)			
HBV	12 (10.0)	24 (12.9)	0.442
HCV	20 (16.7)	22 (11.8)	0.230
None	88 (73.3)	140 (75.3)	0.705
Cardiovascular disease, n (%)	61 (50.8)	83 (44.6)	0.889
Respiratory disease, n (%)	12 (10.0)	20 (10.8)	0.834
Diabetes mellitus, n (%)	34 (28.3)	46 (24.7)	0.484
Cranial nerve disease, n (%)	10 (8.3)	13 (7.0)	0.663
Psychiatric disorder, n (%)	1 (0.8)	2 (1.1)	1.000
Administration of hypnotic drugs, n (%)	7 (5.8)	10 (5.4)	0.865
Administration of antipsychotic drugs, n (%)	1 (0.8)	2 (1.1)	1.000
History of delirium, n (%)	2 (1.7)	2 (1.1)	0.647
ASA class, n (%)			
I	25 (20.8)	52 (28.0)	0.161
II	79 (65.8)	109 (58.6)	0.205
III	16 (13.4)	25 (13.4)	0.979
Preoperative laboratory data, median (range)			
Total bilirubin, mg/dl	0.7 (0.3−2.1)	0.7 (0.2−1.8)	0.740
Albumin, g/dL	4.3 (2.9−4.7)	4.2 (2.9−5.7)	0.160
Prothrombin time, %	94 (50−123)	92 (20−130)	0.897
Creatinine, mg/dl	0.76 (0.48−9.48)	0.76 (0.43−10.39)	0.495
ICG-R15, %	11.6 (0.6−46.0)	12.0 (0.5−56.2)	0.840
Child-Pugh score	5 (5−7)	5 (5−7)	0.688

*ASA*: American Society of Anesthesiologists, *ICG-R15*: indocyanine green retention rate at 15 minutes.

### Surgical characteristics

The size of the tumor, the proportion of patients with multiple tumors, and the frequencies of repeated liver resection, anatomical liver resection, and major hepatectomy did not differ significantly between the groups. The proportion of patients who underwent laparoscopic liver resection was significantly higher in the ramelteon group (63.3% vs. 23.7%, P<0.001). The amount of intraoperative blood loss was significantly lower in the ramelteon group (235 ml vs. 295 ml, P = 0.039), while the operation time did not differ significantly between the groups (**Table 2**).

**Table 2:** Operative characteristics.

	ramelteon n = 120	control n = 186	P-value
Tumor size, mm, median (range)	30 (8−300)	25 (10−120)	0.331
Multiple tumors, n (%)	37 (30.8)	63 (33.9)	0.580
Repeat resection, n (%)	25 (20.8)	56 (30.1)	0.073
Anatomical resection, n (%)	68 (56.7)	90 (48.4)	0.157
Major hepatectomy, n (%)	20 (16.7)	19 (10.2)	0.099
Laparoscopic approach, n (%)	76 (63.3)	44 (23.7)	<0.001
Operation time (min)	318 (71−775)	359 (100−936)	0.152
Blood loss (g)	235 (0−4179)	295 (0−3700)	0.039
Blood transfusion, n (%)	16 (13.3)	23 (12.4)	0.804

### Postoperative outcomes

**Fig 2:**
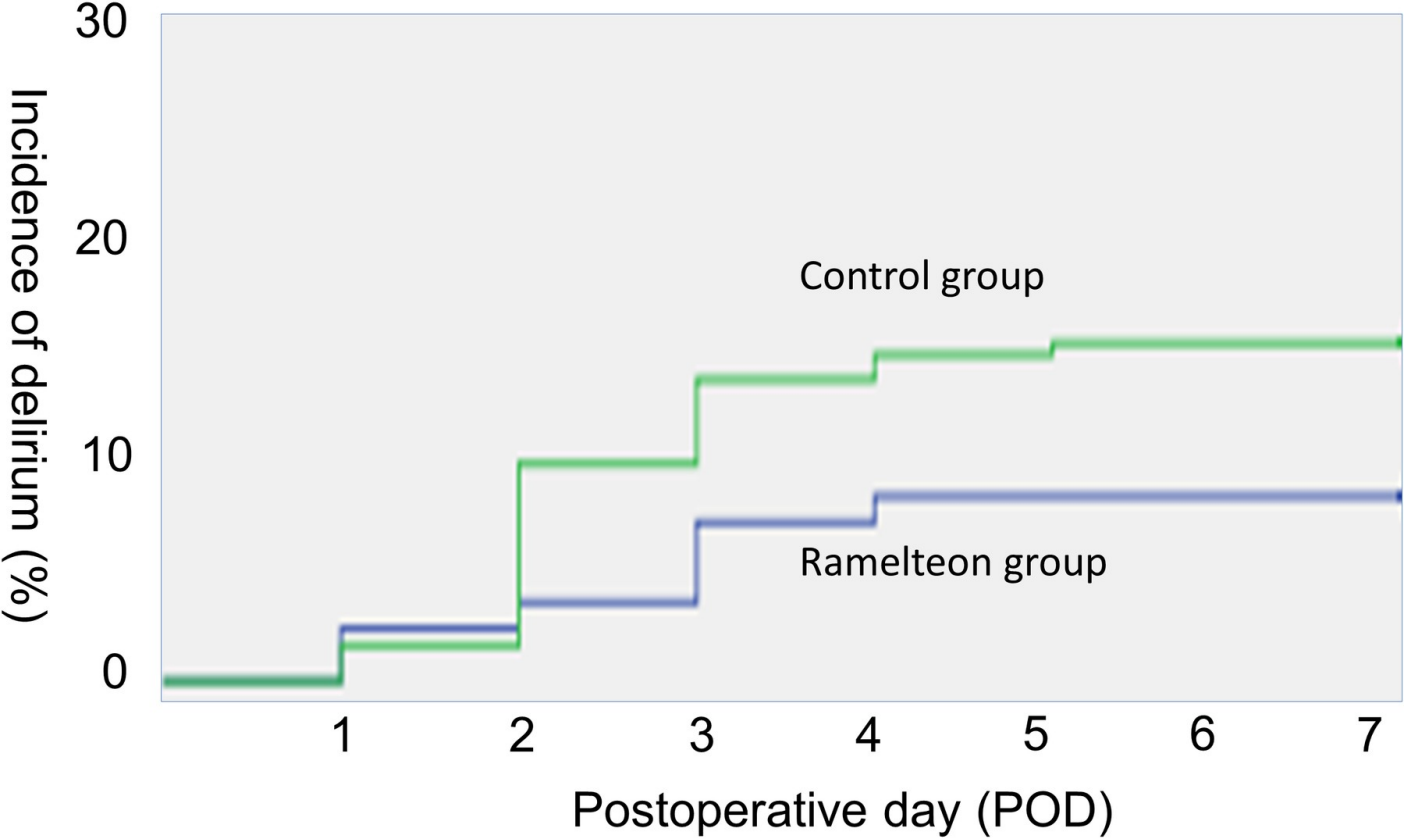
The cumulative incidence of postoperative delirium in each group is shown. The incidence of postoperative delirium was significantly lower in the ramelteon group than in the control group (P = 0.035).

The patients’ postoperative outcomes are summarized in **Table 3**. No patients died within the first 30 days after the operation. Similar incidences of overall and major (Clavien-Dindo ≥IIIa) complications were observed in both groups (35.0% vs. 34.9%, P = 0.992, and 18.3% vs. 12.9%, P = 0.194, respectively). No significant intergroup differences in the incidences of bile leakage (5.8% vs. 9.1%, P = 0.257), surgical site infections (15.8% vs. 17.2%, P = 0.753), or liver failure (9.1% vs. 7.5%, P = 0.609) were observed. The median duration of the patients’ hospital stays was similar in both groups. The incidence of postoperative delirium was significantly lower in the ramelteon group (5.8% vs. 15.1%, P = 0.035). The cumulative incidence of postoperative delirium in each group is shown in **Fig 2**. Factors that interrupted normal postoperative management, type of delirium, POD when the delirium developed, duration of the delirium, and treatment of the delirium were summarized in **Table 4**. There was no significant difference in overall survival (OS) after liver resection between the ramelteon group and the control group (P = 0.524, 3-year OS: 79.3% vs. 83.4%).

**Table 3:** Postoperative outcomes and complications.

	ramelteon n = 120	control n = 186	P value
30-day Mortality, n (%)	0	0	1.000
Postoperative ICU stay, n (%)	8 (6.7)	10 (5.4)	0.640
Overall Complications, n (%)	42 (35.0)	65 (34.9)	0.992
Major Complications (beyond Clavien-Dindo grade IIIa)	22 (18.3)	24 (12.9)	0.194
Ascites	3 (2.5)	6 (3.2)	1.000
Pleural effusion	4 (3.3)	4 (2.2)	0.716
Pneumonia	2 (1.7)	3 (1.6)	1.000
Ileus	2 (1.7)	3 (1.6)	1.000
Bile leakage, n (%)	7 (5.8)	17 (9.1)	0.294
SSI, n (%)	19 (15.8)	32 (17.2)	0.753
Superficial incisional SSI	4 (3.3)	7 (3.8)	1.000
Deep incisional SSI	0	0	1.000
Organ / Space SSI	17 (14.2)	27 (14.5)	0.932
Liver failure, n (%)	11 (9.1)	14 (7.5)	0.609
Postoperative delirium	7 (5.8)	28 (15.1)	0.035
Hospital stay, days, median (range)	9 (4−80)	9 (5−162)	0.893

*ICU*: intensive care unit, *SSI*: surgical site infection.

**Table 4:** Details of delirium.

	ramelteo nn = 7	control n = 28	P value
Factors that interrupted normal postoperative management			
Delay of ambulation, n, (%)	4 (57.1%)	10 (35.7%)	0.401
Delay of oral intake, n, (%)	2 (28.6%)	5 (17.9%)	0.608
Accidental removal of drains, n, (%)	3 (42.9%)	6 (21.4%)	0.340
Trauma caused by delirium, n, (%)	0	1 (3.6%)	1.000
Non-cooperation with medical care, n, (%)	5 (71.4%)	18 (64.3%)	0.218
Type of delirium			
Hyper active type, n, (%)	5 (71.4%)	13 (46.4%)	0.230
Hypoactive type, n, (%)	0	2 (7.1%)	1.000
Mixed type, n, (%)	2 (28.6%)	13 (46.4%)	0.672
POD when the delirium developed			
POD 0–1, n, (%)	2 (28.6%)	3 (10.7%)	0.256
POD 2–3, n, (%)	4 (57.1%)	22 (78.6%)	0.340
POD 4–5, n, (%)	1 (14.3%)	3 (10.7%)	1.000
POD 6-, n, (%)	0	0	
Duration of delirium, days, median (range)	4 (2–7)	3 (1–7)	0.237
Treatment of delirium			
No medication, n, (%)	2 (28.6%)	18 (64.3%)	0.101
Medication			
Haloperidol, n, (%)	4 (57.1%)	8 (28.5%)	0.163
Benzodiazepine, n, (%)	1 (14.3%)	2 (7.1%)	0.500
Other, n, (%)	0	1 (3.6%)	0.800

POD: postoperative day.

### Postoperative outcome of the patients with postoperative delirium

Overall, 35 patients developed postoperative delirium. Postoperative outcomes were compared with 271 patients who did not develop delirium. Postoperative hospital stay was significantly longer in the patients with postoperative delirium (11 days vs. 9 days, P = 0.036). Intensive care unit admissions were more common in the patients who developed postoperative delirium (16.7% vs. 4.8%, P = 0.047). Major complication ≥Clavien-Dindo IIIa was significantly more common in the patients with delirium (36.1% vs. 13.2%, P = 0.004). Postoperative liver failure ≥ISGLS grade B was significantly more common in the patients with delirium (22.9% vs. 6.2%, P = 0.001). Readmission rates within 30 days of discharge were not significantly different (5.7% vs. 3.7%, P = 0635). No significant difference was observed in overall survival after liver resection (P = 0.585). The 3-year survival rate was 82.0% in the patients with delirium and 81.2% in the patients without delirium.

### Univariate analyses of the risk factors for postoperative delirium

Next, analysis of risk factors for postoperative delirium was performed to confirm the preventative effects of ramelteon by multivariate analysis. Thirty-five patients developed postoperative delirium, while 271 patients did not. The results of the univariate analyses of the risk factors for postoperative delirium are summarized in **Table 5**. Regarding baseline characteristics, an age of ≥75 (P<0.001), being male (P<0.001), cardiovascular disease (P<0.001), and an ICG-R15 value of ≥20% (P = 0.010) were identified as significant risk factors for postoperative delirium. As for surgical outcomes, intraoperative blood loss of ≥1000 ml (P<0.001) was found to be significant risk factors for postoperative delirium. The absence of ramelteon treatment was also demonstrated to be a significant risk factor for postoperative delirium (P = 0.017).

**Table 5:** Univariate analysis of risk factors of postoperative delirium.

	delirium n = 35	non-delirium n = 271	P-value
Age, ≥75, n (%)	19 (54.3%)	70 (25.8%)	<0.001
Gender, male, n (%)	33 (94.3%)	176 (64.9%)	<0.001
Alcohol, n (%)	8 (22.9%)	32 (11.8%)	0.069
Cardiovascular disease, n (%)	26 (74.2%)	118 (43.5%)	<0.001
Respiratory disease, n (%)	4 (11.4%)	28 (10.3%)	0.772
Diabetes mellitus, n (%)	12 (34.3%)	68 (25.1%)	0.244
Cranial nerve disease, n (%)	2 (5.7%)	21 (7.7%)	1.000
Viral status, positive, n (%)	10 (28.6%)	68 (25.1%)	0.657
Total bilirubin, ≥1.1mg/dl, n (%)	7 (20.0%)	44 (16.2%)	0.574
Albumin, <3.5g/dL, n (%)	3 (8.6%)	10 (3.7%)	0.176
Prothrombin time, <80%, n (%)	2 (5.7%)	15 (5.5%)	1.000
Creatinine, >1.5mg/dl, n (%)	2 (5.7%)	11 (4.1%)	0.650
ICG-R15, ≥20%, n (%)	13 (37.1%)	50 (18.5%)	0.010
Repeat resection, n (%)	7 (20.0%)	74 (27.3%)	0.357
Major hepatectomy, n (%)	2 (5.7%)	36 (13.3%)	0.201
Laparoscopic approach, n (%)	11 (31.4%)	109 (40.2%)	0.316
Operation time, ≥360min, n (%)	17 (48.6%)	123 (45.4%)	0.722
Blood loss, ≥1000ml, n (%)	13 (37.1%)	37 (13.7%)	<0.001
Non-administration of ramelteon, n (%)	28 (80.0%)	160 (20.0%)	0.017

ASA: American Society of Anesthesiologists, ICG-R15: indocyanine green retention rate at 15 minutes.

### Multivariate analysis of the risk factors for postoperative delirium

The results of the multivariate analysis of risk factors for postoperative delirium are summarized in **Table 6**. An age of ≥75 (P = 0.002), being male (P = 0.032), cardiovascular disease (P = 0.023), blood loss, ≥1000ml (P = 0.001) and the absence of ramelteon treatment (P = 0.046) were found to be independent risk factors for postoperative delirium.

**Table 6:** Multivariate analysis of risk factors of postoperative delirium.

	OR	95% CI	P-value
Age, ≥75	3.69	1.63–8.36	0.002
Gender, male	6.01	1.33–27.23	0.020
Cardiovascular disease	2.67	1.15–6.21	0.023
ICG-R15, ≥20%	2.13	0.90–5.01	0.084
Blood loss, ≥1000ml	4.63	1.92–11.18	0.001
Non-administration of ramelteon	2.64	1.02–6.83	0.046

CI: 95% confidence intervals.

## Discussion

Postoperative delirium is a common complication after liver resection, and it can influence patients’ postoperative prognoses [1, 3]. However, no previous study has examined strategies for preventing postoperative delirium after liver resection. The present study revealed that the administration of ramelteon reduced the incidence of postoperative delirium after liver resection. To the best of our knowledge, this is the first study to examine a strategy for preventing postoperative delirium after liver resection. In the ramelteon group, ramelteon was only administered on the day before surgery and on POD 1 to 3. This intervention was simple and non-invasive. Therefore, the results of this study provide useful information for the perioperative management of liver resection.

This study also investigated the risk factors for postoperative delirium after liver resection. Multivariate analysis revealed that being aged ≥75, being male, intraoperative blood loss of ≥1000 ml, and the absence of ramelteon treatment were independent risk factors for postoperative delirium. Up to now, three previous studies have examined the risk factors for postoperative delirium after liver resection, but no strategy for preventing postoperative delirium in such cases has been described [4–6]. Advanced age was reported to be a risk factor for postoperative delirium in all of the abovementioned studies. On the other hand, male gender was not identified as a risk factor in these studies. However, in large-scale studies of postoperative delirium after surgery involving other organs, male gender was demonstrated to be an independent risk factor for postoperative delirium [15–17]. Another study found that a postoperative hemoglobin level of <11 g/dl, which is similar in nature to intraoperative blood loss of ≥1000 ml, was a risk factor for postoperative delirium [6]. Two previous studies have reported that a low serum albumin level is a risk factor for postoperative delirium [4, 5]. However, no such finding was obtained in the present study. In the current study, univariate analysis suggested that an ICG-R15 value of ≥20% was a risk factor for postoperative delirium, but this was not confirmed in the multivariate analysis. Two previous studies have found that a history of cerebrovascular disorders or hypnotic drug use are risk factors for postoperative delirium, but these factors were not identified as being significantly associated with the risk of postoperative delirium in the present study.

In the current study, the incidence of postoperative delirium after liver resection was 11.4%, which was lower than the figures described in previous reports (17–24.3%) [3–5]. In some previous studies, postoperative delirium was evaluated by psychiatrists using psychiatric diagnosis criteria [18, 19]. On the other hand, in the present study delirium was defined as when normal postoperative care was interrupted due to problems with the level of consciousness or behavior of the patient. Therefore, the previous studies might have included patients with mild delirium. Furthermore, the present study revealed that intraoperative blood loss of ≥1000 ml is an independent risk factor for postoperative delirium after liver resection. In previous studies, the median amount of intraoperative blood loss ranged from 549–605 ml, whereas it was 290 ml in the present study. This fact might have affected the incidence of postoperative delirium.

In this study, all of the patients in the ramelteon group were administered ramelteon. However, it was reported that younger patients rarely develop postoperative delirium [20]. Actually, the youngest patient who developed postoperative delirium in the current study was 73 years old. Thus, it might not be necessary to administer ramelteon to younger patients. Therefore, a prospective study involving older patients would provide more definitive results regarding the preventative effects of ramelteon against postoperative delirium. The incidence of postoperative delirium after liver resection might differ from that seen after other types of surgery because postoperative liver dysfunction can occur after liver resection and the amount of intraoperative blood loss varies among procedures. However, an older age was found to be a general risk factor for postoperative delirium. Therefore, the results of this study might be applicable to other types of surgery.

This study had certain limitations. First, it was a historically controlled study, and the ramelteon group was treated later than the control group. Therefore, the frequency of laparoscopic liver resection was significantly higher in the ramelteon group (63.3% vs. 23.3%, P<0.001). However, there was no significant association between laparoscopic or open liver resection and the development of postoperative delirium. Second, postoperative delirium was not evaluated by psychiatrists using psychiatric diagnostic criteria. Despite these limitations, this study provides useful information about the prevention of postoperative delirium after liver resection. To definitively demonstrate that ramelteon has preventative effects against postoperative delirium, a randomized controlled trial involving older patients is required.

## Conclusions

The results of this study demonstrate the preventative effects of ramelteon against postoperative delirium after liver resection. The administration of 8 mg/day ramelteon on the day before surgery and on POD 1 to 3 might reduce the risk of postoperative delirium after liver resection.

## Supporting information

S1 Data (XLSX)
